# 130. Effects of COVID-19 on a Complex Behavioral Intervention to Improve the Diagnosis and Management of UTI in Nursing Homes

**DOI:** 10.1093/ofid/ofab466.332

**Published:** 2021-12-04

**Authors:** James H Ford, Sally Jolles, Dee Heller, Katie Selle, Daniela Uribe-Cano, Susan Nordman-Oliveira, Jessica Irvine, DaRae Coughlin, Christopher J Crnich

**Affiliations:** 1 University of Wisconsin-Madison, Madison, WI; 2 University of Wisconsin School of Medicine and Public Health, Madison, Wisconsin; 3 Edgewood College, Madison, Wisconsin; 4 University of Wisconsin - Madison, Madison, Wisconsin

## Abstract

**Background:**

Half of all urinary tract infections (UTI) are probably unnecessary. We conducted a cluster-randomized trial in which a toolkit to enhance the diagnosis and treatment of UTIs was introduced in study NHs via usual implementation versus an enhanced implementation approach based on external facilitation and peer comparison reporting.

**Methods:**

Thirty Wisconsin NHs were randomized to each treatment arm in a 1.5:1 ratio. NHs used an online portal to report urine culture and antibiotic treatment data over a 6-month pre-intervention period (Jan-June 2019), a pre-COVID 8-month post intervention period (July 2019-Feb 2020) and an 8-month post-COVID intervention period (Mar-Oct 2020). Study outcomes included urine culture (UC), antibiotic start (AS), and antibiotic days of therapy (DOT) rates per 1,000 resident days. A generalized estimating equation model for panel data was used to assess differences in study outcomes between treatment arms before and after onset of the COVID-19 pandemic. STATA 16.1 was used for all analyses.

**Results:**

A total of 802 UCs (457 pre-COVID, 345 post-COVID), 724 AS (401 pre-COVID, 323 post-COVID), and 6,454 DOT (3553 pre-COVID and 2901 post-COVID) were reported over the 16-month intervention period. No significant differences in the study outcomes were observed during the pre-COVID intervention period, however, UC rates in NHs assigned to the usual care arm of the study increased while those in the enhanced arm declined following onset of COVID-19 (Figure 1). AS and DOT rates followed a similar pattern although the differences between the study arms were not statistically significant.

Figure 1. Post Implementation Periods

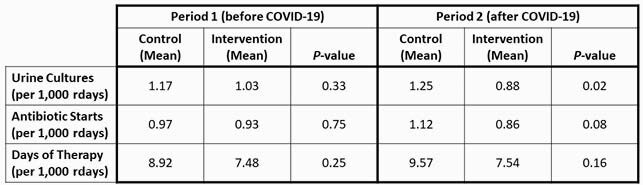

**Conclusion:**

Our findings suggest that NHs assigned to usual implementation regressed in their diagnosis and treatment of UTIs during the COVID-19 pandemic while those receiving external facilitation and peer comparison reports were more resilient to the effects of COVID-19.

**Disclosures:**

**All Authors**: No reported disclosures

